# Comorbid Illness, Bowel Preparation, and Logistical Constraints Are Key Reasons for Outpatient Colonoscopy Nonattendance

**DOI:** 10.1155/2016/2179354

**Published:** 2016-07-11

**Authors:** Deepti Chopra, Lawrence C. Hookey

**Affiliations:** Gastrointestinal Diseases Research Unit, Queen's University, Kingston, ON, Canada K7L 2V7

## Abstract

*Background.* Colonoscopy nonattendance is a challenge for outpatient clinics globally. Absenteeism results in a potential delay in disease diagnosis and loss of hospital resources. This study aims to determine reasons for colonoscopy nonattendance from a Canadian perspective.* Design.* Demographic data, reasons for nonattendance, and patient suggestions for improving compliance were elicited from 49 out of 144 eligible study participants via telephone questionnaire. The 49 nonattenders were compared to age and sex matched controls for several potential contributing factors.* Results.* Nonattendance rates were significantly higher in winter months; the OR of nonattendance was 5.2 (95% CI, 1.6 to 17.0, *p* < 0.001) in winter versus other months. Being married was positively associated with attendance. There was no significant association between nonattendance and any of the other variables examined. The top 3 reasons for nonattendance were being too unwell to attend the procedure, being unable to complete bowel preparation, or experiencing logistical challenges.* Conclusions.* Colonoscopy attendance rates appear to vary significantly by season and it may be beneficial to book more colonoscopies in the summer or overbook in the winter. Targets for intervention include more tailored teaching sessions, reminders, taxi chits, and developing a hospital specific colonoscopy video regarding procedure and bowel preparation requirements.

## 1. Introduction

Colonoscopy cancellations and nonattendance are universal problems in the realm of gastroenterology. Nonattendance rates have reached an average of 14% in Europe with similar numbers in the United States [1, 2]. The lack of forewarning that accompanies missed appointments leaves insufficient time to book new colonoscopies, resulting in a potential delay in disease diagnosis and loss of finite hospital resources.

There are a myriad of documented reasons for colonoscopy nonattendance including socioeconomic, cultural, patient, physician, and organizational barriers [1–10]. Several studies have demonstrated that factors such as fear of positive findings, poor understanding of the procedure, anxiety about the procedure, and absence of symptoms all play a role [1–4]. Farzin-Moghadam et al. state that patients of gastroenterologists are more likely to be adherent than those of surgeons [5]. Others promote the notion of apathy suggesting that patients forget their appointments or forget to cancel [1, 6]. Patient adherence to scheduled outpatient colonoscopy has been found to improve as the week progresses and worsen in the afternoons, highlighting the influence of timing on compliance [7]. Bowel preparation schedules can also impact colonoscopy completion rates by causing a loss of working hours or sleep disturbance [8, 9]. Difficulty finding transportation after finishing colonoscopy may also be a consideration for certain individuals [2]. Rates of nonattendance do not appear to differ by age or sex; however, low-income minorities face organizational barriers when attempting to obtain and complete screening colonoscopy in the United States [10].

Solutions have been proposed to ameliorate the challenges with colonoscopy completion. Patient education and reminder phone calls significantly reduce appointment cancellations [6, 11–15]. Ayanian et al. suggest that primary care providers should also be a target for reminders [16]. Patient navigation and outreach services have been shown to improve colonoscopy attendance, although the precise features of navigation that aid appointment maintenance are yet to be determined [17, 18]. Denberg et al. suggest that mailed brochures can increase patient adherence while others encourage overbooking [19, 20]. Peer coach support appears to promote colonoscopy attendance for patients who regularly fail to keep appointments [21]. Marital status may also affect attendance and Laiyemo et al. propose that married patients are more likely to attend [22]. Overall, the implementation of such practices is highly center-specific and often requires additional financial and employee support that is not available at many institutions.

This study aims to determine the reasons for colonoscopy cancellations and nonattendance in the outpatient setting and is the first to do so from a Canadian perspective. Moreover, there is a paucity of literature grounded in root cause analysis and our study is one of the few that is geared towards prospectively eliciting patient experience and perspectives on the issue of nonattendance through telephone surveys, as opposed to pure retrospective chart review. Knowledge of the current causes of missed colonoscopies will enable the implementation of more tailored quality control interventions that will maximize resource utilization and enhance patient care.

## 2. Methods

Eligible study participants were patients from Hotel Dieu Hospital requiring colonoscopy who cancelled the appointment within 3 days of their scheduled date or did not attend on the day of the procedure. Reasons for colonoscopy nonattendance were elicited through telephone survey and a case-control design was used to compare attenders and nonattenders for eight selected predictor variables. The investigator D. Chopra performed the telephone survey over a 1.5-year time period, including a fiscal year at Hotel Dieu Hospital ±0.5 years to capture a maximal amount of consecutive nonattenders over the study period (July 2012–January 2014). Ethics approval was obtained from the Queen's University Health Sciences Research Ethics Board and consent for chart review of colonoscopy attenders was acquired upon admission to hospital. Three days was selected as the cut-off point for nonattendance as this represents the time required for bowel preparation and the period in which there is an inability to refill colonoscopy slots. Patients receiving upper endoscopy alone were excluded from the study but patients scheduled for colonoscopy or combined colonoscopy and upper endoscopy were enrolled. The age range of the included sample was 19–90 years.

A questionnaire was developed by study investigators based on detailed literature review and understanding of the current challenges with colonoscopy appointment adherence. Key factors which recurred in the literature and an informal survey of colonoscopists at our center were used to direct the specific components included in the questionnaire. Moreover, in order to minimize bias, an open-ended question (question number 9) was used to obtain patient directed, spontaneous answers regarding the reason why they could not attend their appointment (*see supplemental data *in Supplementary Material available online at http://dx.doi.org/10.1155/2016/2179354). Only after this information was collected, were closed-ended questions used to determine if there were any other potential contributors (*see question number 11 in supplemental data*). The study was piloted with 10 participants and investigator feedback was incorporated into the survey tool. The final questionnaire comprised of demographic data as well as questions regarding reasons for nonattendance and whether or not the procedure was rescheduled and suggestions for assisting patients with keeping appointments (*see supplemental data*).

Age and sex matched controls were randomly selected from patients who had attended their first colonoscopy in the last 1.5 years, over the same time period as cases (July 2012–January 2014). Two controls of the same age and sex were selected. Cases were compared for the following factors via chart review: first colonoscopy, prior clinic visit, screening versus symptomatic, distance from Hotel Dieu Hospital, timing of the colonoscopy (winter months versus other months, AM versus PM), type of bowel preparation, and marital status. For the purposes of our study analysis, indication for colonoscopy was dichotomized to screening versus symptomatic. In this case, screening colonoscopy also includes surveillance colonoscopy and a patient presenting for colonoscopy secondary to overt gastrointestinal clinical signs or symptoms such as bleeding, abdominal pain, diarrhea, nausea, or vomiting was classified as symptomatic. Distance from Hotel Dieu Hospital was categorized as 1–5 km, 6–10 km, 11–15 km, 16–20 km, and >20 km. In terms of timing of colonoscopy, groups were compared for winter (December, January, February, and March) versus other months of the year and am [08:00–12:00] versus pm [12:00–16:00] appointments. Bowel preparations of either sodium picosulfate plus magnesium citrate (P/MC) or polyethylene glycol solution (PEG) were also compared between cases and controls.

### 2.1. Data Analysis

Patient demographic data was collated, assessed descriptively, and tabulated to determine the underlying distribution. Qualitative data was collated and analyzed* post hoc*. Common themes of patient reported reasons for missed colonoscopies and suggestions for improvement were elicited and responses were categorized according to these themes.

Cases were matched with controls on sex and exact year of age. Although 50 cases were enrolled, one 91-year-old patient was excluded because an exact match could not be found for her leaving 49 cases and 98 controls in this analysis. Since we had several prespecified exposures of interest rather than one single primary exposure of interest, we defined the sample size so that any of our exposures with at least 25% prevalence would have adequate power to detect a large effect size. Assuming that the exposure rate was at least 25%, our sample size of 49 cases and 98 matched controls achieves at least 80% power at a two-sided alpha = 0.05 if the conditional odds ratio of exposure between the cases and controls is greater than 3 or less than 1/3.

The Cochran-Mantel-Haenszel method was used to estimate *p* values and conditional odds ratios for the association between missing an appointment and our selected predictor variables, while stratifying for age and sex. The odds ratio for distance from Hotel Dieu Hospital was calculated per 10 kilometers. All analyses were performed using SAS version 9.4 (SAS Institute Inc., Cary, NC, USA).

## 3. Results

### 3.1. Response Rate

2941 colonoscopies were conducted over the 2013-2014 fiscal year at Hotel Dieu Hospital. A total of 280 patients did not attend their scheduled procedure in that time period. We attempted to contact all nonattenders over a period of 1.5 years until the target sample size of 50 questionnaire responses was achieved. A total of 144 patients were approached. Seventy-six were unreachable, 3 patients' numbers were not in service, 15 patients declined to participate, 50 patients consented to the telephone questionnaire, and 49 patients were matched with two controls on sex and exact age resulting in a response rate of 35%.

### 3.2. Patient Demographics

**Figure 1:**
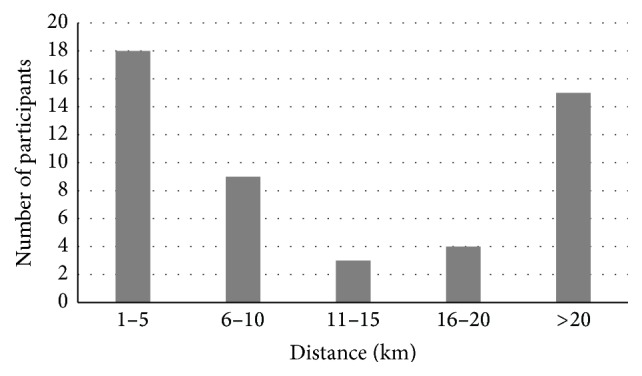
Distance of patients from Hotel Dieu Hospital.

Nonattender characteristics are summarized in Table 1. The majority of participations were >50 years old. The mean distance of patients from Hotel Dieu Hospital was 20 kilometers. Thirty-seven percent of participants lived within 5 kilometers of the hospital and 31% lived ≥20 kilometers away (Figure 1). Fifty-seven percent of participants who cancelled or did not attend their colonoscopy were female. Forty-seven percent of nonattenders were married. Less than one-third of patients reported that the scheduled procedure would have been their first colonoscopy. At least half the patients had a clinic visit prior to the scheduling of their colonoscopy. Screening colonoscopy was the indication for the procedure in a majority of cases. Seventy percent of nonattenders were successfully able to reschedule their colonoscopy. Of those who rescheduled their colonoscopy, 70% actually attended their subsequent appointment.

### 3.3. Top Reasons for Missed Colonoscopies

**Figure 2:**
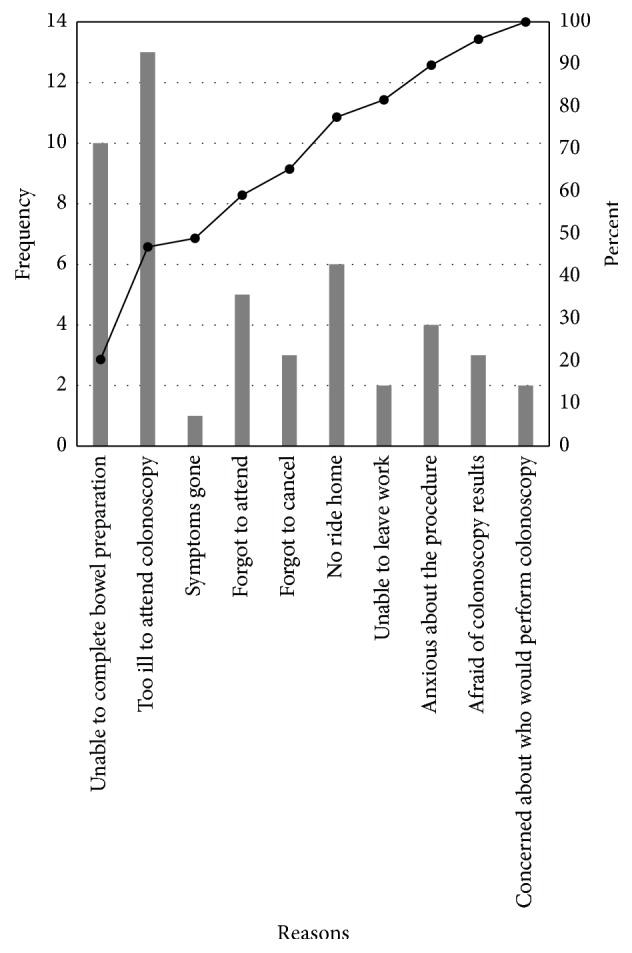
Top reasons for missed colonoscopies.

Based on our predesigned questionnaire, the top 3 reasons for missed colonoscopies were being too ill to attend the colonoscopy (27%), being unable to complete bowel preparation (20%), and not having transportation after finishing procedure (12%). Apathy and anxiety were the next most common causes of nonattendance (Figure 2). Patient reported reasons correlated very well with the responses elicited via closed-ended questions from our survey. Again, a majority of patients recalled that they were too ill to attend their colonoscopy. They reported feeling unwell with symptoms unrelated to their gastrointestinal health, for example, upper respiratory tract infection or flare of a preexisting medical condition rendering them unable to leave their home. Alternatively, some patients required transfer to inpatient care upon arrival to their procedure (*N* = 2). Ten nonattenders (20%) experienced difficulties related to bowel preparation. Patients stated that they were unable to complete their bowel preparation, felt too ill or weak from the preparation, or completed the preparation ineffectively. Many encountered logistical challenges such as being unable to receive time off work, being unable to obtain transportation, or facing extreme weather conditions. Several patients did also forget to attend their appointment or cancel in advance. A minority of patients reported clerical errors or confusion surrounding their procedure date or time. Anxiety and concerns about who would be conducting the colonoscopy were contributors to nonattendance for some patients. Other factors such as funerals or being the sole caregiver to another individual played a role in missed colonoscopies for 4% of patients.

### 3.4. Comparison of Nonattenders to Age and Sex Matched Controls

Nonattendance rates varied significantly by calendar month. In particular, the nonattendance rate was significantly higher in winter months (December, January, February, and March) compared with other months; the odds ratio of nonattendance was 5.2 (95% CI, 1.6 to 17.0, *p* < 0.001). The conditional odds ratio of married people being nonattenders was 0.41 (95% CI, 0.19 to 0.88, *p* = 0.004) compared to that of nonmarried people. There was no suggestion of an association between nonattendance and any of the other variables examined: first colonoscopy (*p* = 0.89), clinic visit prior to colonoscopy (*p* = 1.00), screening versus symptomatic (*p* = 0.63), distance from hospital (*p* = 0.47), or AM versus PM (*p* = 0.49) (Table 2). The difference between the types of bowel preparation used in nonattenders versus attenders was not statistically significant (*p* = 0.1). 54% of nonattenders were prescribed sodium picosulfate plus magnesium citrate (P/MC), 22% were prescribed polyethylene glycol solution (PEG), and one case was unknown. In the attenders group, 69% used P/MC and 32% used PEG.

### 3.5. Patient Suggestions for Improving Colonoscopy Attendance

Table 3 highlights the key suggestions patients provided regarding methods to improve colonoscopy attendance. The majority of patients (53%) were content with the current process of obtaining and completing a colonoscopy. For those who felt improvement was warranted, their suggestions focused on enhanced communication. Twenty-two percent of patients suggested that better communication would have helped them to keep their scheduled appointment. Of those 22%, over half reported that they would have liked more detailed communication from their physician, including clearer explanations of what the procedure involves and what adequate bowel preparation entails. The remaining half expressed concerns about appointment scheduling or cancellations when speaking to the receptionist, and some felt their nurse educator could have been more thorough with bowel preparation teaching.

Another theme that emerged was the notion of reminders. Fourteen percent of patients felt that various forms of reminders would have been helpful in enabling them to maintain their appointment. Some suggested reminder phone calls ranging from 1 to 5 days prior to the colonoscopy. Others suggested reminder emails including instructions on how to complete the bowel preparation. One individual reported that a reminder to complete prerequisite investigations would have been helpful. Ten percent of patients provided additional suggestions including minimizing the time between clinic visits and procedure dates, providing transportation for patients who have none, and booking appointments in the spring/summer when the weather is less severe.

## 4. Discussion

Outpatient colonoscopy nonattendance rates at Hotel Dieu Hospital are 8–10% annually. Current literature has outlined numerous reasons for poor attendance; however, there is a lack of consensus on the greatest contributing factors. This study has highlighted several novel reasons for missed colonoscopy appointments in the Canadian setting. Previous research has proposed apathy, clerical errors, anxiety, and resolution of symptoms as key reasons for absenteeism [1–3, 6]. Contrary to these results, our study found that the primary reason for nonattendance was illness from preexisting medical comorbidities or acute infection unrelated to gastrointestinal disease. Twenty-seven percent of patients were too unwell to leave their house and attend their colonoscopy. Perhaps there needs to be a greater emphasis on informing patients to call in advance and cancel their appointment if they are ill. Moreover, Badurdeen et al. suggest that the timing of appointments can affect compliance, with attendance decreasing in the morning and as the week progresses [7]. We did not find any difference in rates of attendance between morning and afternoon appointments or based on distance from the clinic. However, there was a statistically significant difference between attendance rates based on month of the year, with nonattendance increasing from December to March. Hence, it may be important to book more colonoscopies in the spring and summer months in order to optimize colonoscopy completion. Equally, it may be wise to overbook during the winter season.

This study was in agreement with the literature in revealing that bowel preparation and logistical constraints were crucial barriers to appointment attendance [2, 8, 9]. Many patients reported difficulty in completing bowel preparation or were too symptomatic from the preparation to attend their colonoscopy. However, interestingly, there was no difference between the types of bowel preparations used in attenders and nonattenders. Moreover, 16% did not have transportation before/after procedure, faced extreme weather conditions, or did not have the financial or community support required to obtain transportation. Logistical limitations seem to be an underappreciated obstacle to colonoscopy completion and may be a feature that is specific to the Canadian climate. Our center has already begun to address this issue by implementing a new policy where patients are observed for 6 hours after procedure and provided with a taxi chit to travel home if needed. Moreover, this study corroborates findings from Laiyemo et al. which indicate that there may be a trend towards marital status having a protective effect on maintaining appointments and social support systems likely positively influence adherence [22].

With respect to solutions for colonoscopy nonattendance, our study substantiates current knowledge that patient reminders may be an integral means of improving appointment maintenance [6, 13–15]. Participants reported that reminders in the form of telephone calls and emails would have helped them to attend. The preferred timing of reminders ranged from 1 to 5 days prior to the procedure and some patients requested reminders on when and how to complete bowel preparations, in addition to the actual appointment time. Further research is required to delineate the optimal timing and mode of administering reminders to patients.

To date, improved communication with patients has not been heavily emphasized as a method of increasing attendance. We found that ineffective communication was a cause for concern in our study population, as they felt there was a lack of clear explanation about the procedure and bowel preparation, despite teaching from our nurse educator. Sixteen percent of participants wanted more detailed explanations from their physician and nurse educator. A minority of patients also had concerns about clerical errors and interactions with the receptionist. Hence, there is still room for the health care team to advance patient education and communication strategies. As suggested by Arabul et al., developing a hospital specific colonoscopy video may be a cost-effective and efficient means of disseminating information about the colonoscopy and bowel preparations and may mitigate some of the associated anxieties and fears [23]. The video could be viewed in clinic with a nurse educator present to answer any potential questions and could theoretically also be mailed to patients as a DVD or USB drive for reviewing at home.

In terms of our study limitations, there is an inherent challenge in communicating with a target population of nonattenders. Our response rate of 35%, despite multiple attempts at contact, illustrates this point. We anticipated difficulty in reaching a population of individuals who were unable to make their scheduled appointments; however, our response rate was comparable to UK data which achieved a 30% response from patients contacted via telephone [1]. Nonetheless, we reached our target number of cases and controls. In order to offset the recall bias inherent to retrospective data collection, we contacted participants within 3 days of their missed appointment.

There remains a steady volume of colonoscopy cancellations and absenteeism nationally and globally. This is the first Canadian study to use telephone interviews to assess patient reasons for nonattendance, rather than solely obtaining objective information from medical charts. By contacting nonattenders over the course of 1.5 years, we have been able to elucidate novel reasons for missed procedures and reveal tangible areas for improvement that will serve to increase completion rates, optimize resource utilization, and enrich patient care.

## Supplementary Material

The supplemental data provides the questionnaire designed by study investigators to elicit key information from participants via telephone survey.

## Figures and Tables

**Table 1:** Nonattender demographics.

Characteristic	Mean (standard deviation) [range]
Age (years)	60 (12) [19–90]
Distance from HDH^*∗*^ (km)	20 (31) [1–182]

Characteristic	Frequency (percent)

Sex:	
Male	21 (43)
Female	28 (57)
Married	23 (47)
First colonoscopy	15 (31)
Prior GI^+^ clinic visit	27 (55)
Indication for colonoscopy:	
Screening	35 (71)
Symptomatic	15 (31)

^+^Gastroenterology.

^*∗*^Hotel Dieu hospital.

**Table 2:** Comparison of nonattenders to age and sex matched controls.

Variable	Univariate odds ratio	95% CI	*p* value
First colonoscopy (yes/no)	1.05	0.48–2.31	0.89
Prior clinic visit (yes/no)	1.00	0.49–2.02	1.00
Indication (screening versus symptomatic)	0.85	0.42–1.70	0.63
Distance from HDH (per 10 km)	0.97	0.88–1.06	0.47
Winter month (December, January, February, and March) versus other months of the year	5.20	1.59–17.02	<0.001
am [08:00–12:00] versus pm [12:00–16:00]	0.78	0.39–1.57	0.49
Married versus nonmarried	0.41	0.19–0.88	0.004

**Table 3:** Patient suggestions for improving colonoscopy attendance.

Suggestion	Frequency (percentage)
No need for improvement	26 (53)
Communication:	11 (22)
(i) Receptionist	3 (6)
(ii) Physician	6 (12)
(iii) Nurse providing bowel preparation teaching	2 (4)
Reminders	7 (14)
Other	5 (10)

## Appendix

See Tables 1, 2, and 3 and Figures 1 and 2.
