# Supplementary material for: Comorbid Illness, Bowel Preparation, and Logistical Constraints Are Key Reasons for Outpatient Colonoscopy Nonattendance
Source: Can J Gastroenterol Hepatol. 2016 Jul 11;2016:2179354. doi: 10.1155/2016/2179354 (PMC4958479; doi:10.1155/2016/2179354)
Supplement: Supplementary file 1 — The supplemental data provides the questionnaire designed by study investigators to elicit key information from participants via telephone survey. [file 2179354.f1.pdf]

Queen's UNIVERSITY

Data Collection Form

Reasons for Colonoscopy Cancellation and Non-Attendance in Kingston, Ontario

Patient Name:

1) How old are you? ____________ years
   - 20-30 years old ___
   - 31-50 years old ___
   - 51-70 years old ___
   - 71-90 years old ___
   - 91-110 years old ___

2) Sex: ___________________________

3) How far do you live from Hotel Dieu Hospital?
   - 1-5 km ___
   - 6-10 km ___
   - 11-15 km ___
   - 16-20 km ___
   - >20 km ___

4) Where do you live?

____

5) What was the date of your scheduled colonoscopy? ___________________________

6) Was that your first colonoscopy?

- Yes ________ No ________

7) Did you have an appointment at the clinic before your colonoscopy appointment?

- Yes ________ No _________
- If yes, when was it?

  ____

8) Why did you need a colonoscopy?

____

- Screening ____________ or Symptomatic ______________

9) What prevented you from attending your colonoscopy?

____

10) Did you reschedule your missed colonoscopy?

- Yes ________ No _________
- Rescheduled for ___________________________

11) Did any of the following reasons affect your ability to attend your colonoscopy?

Potential Reason for Missed Colonoscopy	Yes	No
You were unable to complete your bowel preparation		
You were too sick to attend your colonoscopy		
Your symptoms were gone		
You forgot to attend your colonoscopy		
You forgot to cancel your appointment		
You did not have a ride home		
You were unable to leave work		
You were anxious about the procedure		
You were afraid of the results of the colonoscopy		
You were concerned about who would be conducting the colonoscopy		

a) Please rank your top reason from the list above?

Top Reason: _______________________________________

12) What do you think the medical team could have done to help you keep your colonoscopy appointment?

____
